# Laser Powder Bed Fusion of Pure Tungsten: Effects of Process Parameters on Morphology, Densification, Microstructure

**DOI:** 10.3390/ma14010165

**Published:** 2020-12-31

**Authors:** Junfeng Li, Yunxiao Wu, Bokang Zhou, Zhengying Wei

**Affiliations:** The State Key Laboratory for Manufacturing Systems Engineering, Xi’an Jiaotong University, Xi’an 710049, China; zhou1015@stu.xjtu.edu.cn (B.Z.); wuyunxiao666@stu.xjtu.edu.cn (Y.W.)

**Keywords:** laser powder bed fusion, microstructure, morphology, pure tungsten, relative density

## Abstract

Tungsten has been widely used in many industrial fields due to its excellent properties. However, owing to its characteristics of inherent brittleness at room temperature and high melting point, it is difficult to prepare tungsten parts with high complexity via traditional methods. In the present work, tungsten samples were prepared by laser powder bed fusion. The influence of each process parameter including laser power, scanning speed, and hatch spacing on the surface morphology, densification, and microstructure of tungsten samples was systematically investigated. The results showed that the use of the appropriate parameters, especially high laser power, can effectively improve the surface quality and obtain a dense surface. The tungsten samples with a relative density of 98.31% were obtained with optimized parameter combinations: a laser power of 300 W, scanning speed of 400 mm/s, and hatch spacing of 0.08 mm. Compared with scanning speed and hatch spacing, the laser power had a more obvious influence on the relative density. Additionally, for the grain morphology by microstructure inspection, elongated curved grains gradually transformed into fine straight columnar grains as the scanning speed increased. The hatch spacing would change the grain morphology slightly but had no significant effect on the grain size.

## 1. Introduction

Tungsten (W), the metal with the highest melting point (3695 K), has been widely used in many fields including aerospace, electronics, and medical devices due to its excellent properties such as high melting point, high density, good thermal conductivity, and low thermal expansion coefficient [1,2,3]. W is also considered to be a promising candidate plasma-facing material for the divertor devices in the nuclear industry because of its characteristics of sputtering resistance and low tritium retention [4,5]. However, tungsten presents brittleness at room temperature due to its high ductile-brittle transition temperature (DBTT) [6]. Therefore, it is difficult to use conventional methods to manufacture tungsten parts with high complexity.

Laser powder bed fusion (LPBF), which applies a high-energy laser beam to melt pre-deposited metallic powder layer by layer according to the computer-aided design data, has been widely used in the preparation of many kinds of materials, such as steel, titanium, aluminum, and nickel [7]. To date, LPBF has shown great advantages and potential in the manufacture of complex parts [8,9]. In recent years, the application of high-power laser makes LPBF possible to manufacture materials with a high melting point directly, which has aroused the interests of people from industrial and research fields. Zhou et al. [10] obtained pure tantalum samples with high strength and ductility by LPBF. Wang et al. [11] investigated the application of LPBF to pure molybdenum and obtained high dense molybdenum with a relative density of 99.1% by using spherical powder. However, compared to refractory metals such as molybdenum and tantalum, the direct process of tungsten by LPBF is more challenging. Enneti et al. [12] discussed the effect of hatch spacing and scanning speed on the densification of pure tungsten of LPBF. However, they prepared tungsten samples with a relative density of only 75%. Zhou et al. [13] investigated the balling issues in tungsten of LPBF, and manufactured tungsten samples with a relative density of 82.9% by applying a re-melting strategy. Wang et al. [14] analyzed the influence of spherical and polyhedral tungsten powder on the morphology and densification of tungsten in LPBF and found that spherical particles could promote the densification process. They obtained tungsten samples with a relative density of 96% by using spherical pure tungsten powder. Wen et al. [15] analyzed the effect of scanning speed on the surface morphology, densification, and microstructure of the tungsten of LPBF and manufactured tungsten samples with a relative density of 98.71%. Xiong et al. [16] prepared pure tungsten samples with a relative density of 98.1% by optimizing the laser power and scanning speed. Meanwhile, Tan et al. [17] investigated the effect of different linear energy densities (the ratio of laser power to scan speed) on relative density and obtained tungsten samples with a relative density of 98.5%, without discussing the role of a single factor in the LPBF process of tungsten. Similarly, Guo et al. [18] discussed the influence of five volumetric energy densities on densification, microstructure, and mechanical properties, and tungsten samples with a relative density of 98.4% were prepared by LPBF. Also, Sidambe et al. [19] manufactured pure tungsten samples with a relative density of 98% by optimizing the volumetric energy density and discussed the influence of volumetric energy density on microstructure and texture. Ivekovic et al. [20] performed investigations on the effects of different volumetric energy densities on the relative density of pure tungsten and obtained tungsten samples with a relative density of 97.1%. Gu et al. [21] discussed the effect of scanning strategy on the surface morphology, microstructure, and properties of pure tungsten. Simultaneously, the effects of process parameters on tungsten thin-wall parts were investigated by Xie et al. [22] and Wu et al. [23]. In summary, although a series of investigations on tungsten of LPBF that have been carried out, most of them focus on the effect of a single factor or energy density on the relative density and microstructure of tungsten. However, according to previous studies, energy density is insufficient to completely determine the final relative density and resultant microstructure of samples fabricated by LPBF [24,25]. Therefore, it is still necessary to systematically discuss the influence of each factor including laser power, scanning speed, and hatch spacing on the surface morphology, relative density, and microstructure of pure tungsten in LPBF. Meanwhile, LPBF is a process with multiple factors. An investigation on the most dominant factor under several factors has not been carried out in pure tungsten of LPBF.

Therefore, in the present work, the effect of each process parameter on the surface morphology, relative density, and final microstructure was systematically investigated. The surface morphology and the formation of defects under different process parameters were characterized and analyzed in detail. The significance of the influence of each process parameter on the relative density is discussed through the analysis of variance (ANOVA). Moreover, the influence of process parameters on grain morphology was discussed, and quantitative analysis of solidification grain size under different scanning speed and hatch spacing was conducted based on electron backscattered diffraction (EBSD) analysis.

## 2. Materials and Methods

### 2.1. Tungsten Powder Feedstock

**Figure 1:**
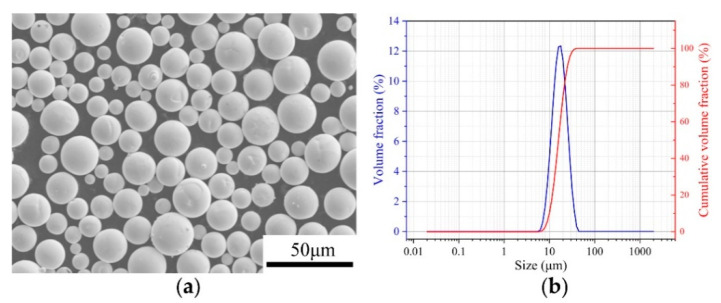
(**a**) SEM morphology of tungsten powder; (**b**) particle size distribution.

The feedstock used in this experiment is pure tungsten powder with good spherical morphology, as presented in Figure 1a, which is beneficial for the flowability of powder and uniformity of the resultant powder layer. As shown in Figure 1b, the particle size distribution of tungsten powder can be observed, and the detailed information of powder size is D10 = 10 μm, D50 = 15.87 μm, and D90 = 24.86 μm.

### 2.2. Experimental Methods and Parameters

**Figure 2:**
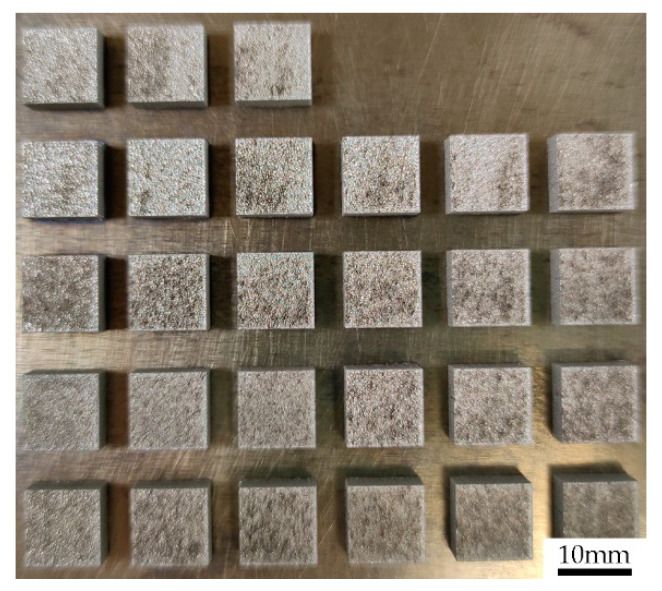
LPBF-processed tungsten samples.

**Figure 3:**
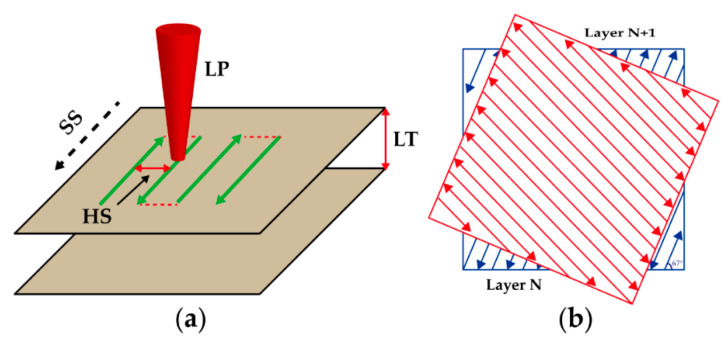
(**a**) Illustration of process parameters; (**b**) scanning strategy used in this work.

Tungsten samples with dimensions of 10 × 10 × 5 mm^3^ were prepared by the SLM 280HL (SLM solutions, Lübeck, Germany), as shown in Figure 2. During the LPBF process, high-purity argon was used as a protective gas and the oxygen content in the chamber was kept below 300 ppm. Also, the preheating temperature of 316 L stainless steel substrate was set at 423.15 K in order to reduce the tungsten samples warping tendency from the substrate. Table 1 gives the process parameters including laser power (LP), scanning speed (SS), hatch spacing (HS) of the LPBF used in this experiment, and the measured relative density of as-built tungsten samples. In addition, the layer thickness used in this work was kept at 0.03 mm. Figure 3a shows the illustration of process parameters in LPBF, and Figure 3b gives the schematic diagram of the scanning strategy adopted in this work. As can be seen from Figure 3b, a zigzag scanning strategy was used for a single layer, with a rotation of 67° between adjacent layers. Besides, an analysis of variance (ANOVA) was carried out by using Minitab 17.0 software to assess the main effects of the process parameters on the relative density of as-built tungsten samples.

### 2.3. Materials Characterization

All tungsten samples were removed from the substrate via wire electrical discharge machining. Subsequently, the samples were cleaned using an ultrasonic cleaning machine and then dried. The top surface morphology of tungsten samples was observed by Scanning electron microscope (SEM, S-4800, Hitachi, Tokyo, Japan). For metallographic observation, tungsten samples were prepared according to the standard metallographic procedure. The cross-section of samples was ground by SiC sandpaper with 400#, 600#,800#,1000#,1200#, and polished using diamond suspension with 2.5 μm and 0.5 μm. For microstructure observation, polished samples were etched with a mixed solution of H_2_O, NaOH, and K_3_[Fe(CN)_6_] in a ratio of 10:1:1. Subsequently, the microstructure were observed using an Optical microscope (MA-200, Nikon, Tokyo, Japan). The relative density of as-built tungsten samples was measured according to the Archimedes method, and the average values were calculated based on the measured results three times. For the further analysis of grain, electron backscattered diffraction analysis was performed by EBSD systems (Oxford Instruments, Oxford, England) with a step size of 1.5 μm. The Channel 5 software was used to analyze the obtained EBSD data.

## 3. Results and Discussion

### 3.1. Surface Morphology

**Figure 4:**
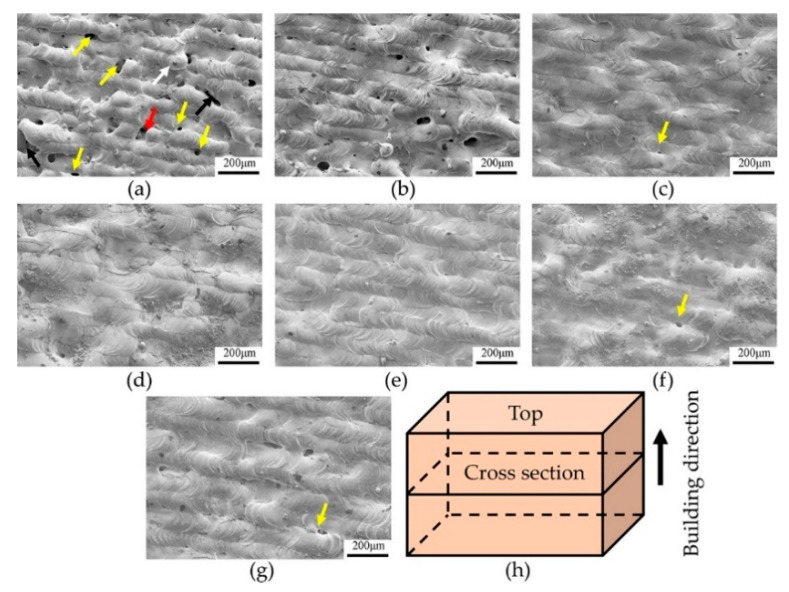
The top surface morphology of tungsten samples under different process parameters. (**a**) T5, (**b**)T14, (**c**)T23, (**d**)T20, (**e**)T26, (**f**)T22, (**g**)T24, and (**h**) illustration of sample orientation.

During the LPBF process, single tracks are formed due to the continuous irradiation of laser to the pre-deposited powder layer. Subsequently, the single-layers are built as a result of the combination of multiple single-tracks. Finally, the parts are manufactured layer-by-layer. Therefore, it can be concluded that it is very important to ensure each layer has good surface quality for the manufacturing of the final part with high relative density due to the inherent characteristics of layered accumulation of LPBF. The surface morphology is directly determined by process parameters including laser power, scanning speed, and hatch spacing. Thus, it is necessary to investigate the influences of the process parameters on single-layer morphology. Figure 4 shows the top surface morphology of as-built tungsten samples under different process parameter combinations. Figure 4a–c depicts the surface morphology under different laser power when the applied scanning speed was 300 mm/s and hatch spacing was 0.08 mm. It can be seen that porous and irregular surface gradually becomes dense and flat with laser power increased from 200 W to 300 W. In Figure 4a, the surface defects can be classified into four types including gaps (red arrow), humps (white arrow), cavities (black arrow), and pores (yellow arrow). With the applied laser power increased to 250 W, it can be found in Figure 4b that the surface defects including gaps, bulges, and cavities disappeared, and only some pores remained. Furthermore, once the applied laser power increased to 300 W, the remaining pores almost disappeared, as shown in Figure 4c. This phenomenon can be explained by different states of tungsten melt caused by different heat inputs due to the variation of laser power. The surface tension and dynamic viscosity of tungsten melt can be described by the following equations [26]:

σ(T) = 2.48 − 0.31 × 10^−3^(T − T_m_) T ≥ 3695 K, (1)

µ(T) = 0.16 × 10^−3^ exp (3.9713T_m_/T) T ≥ 3695 K, (2)

where, the σ is surface tension, N/m, T is the temperature of tungsten melt, K, T_m_ is the melting point temperature of tungsten material, K, and µ is the dynamic viscosity of tungsten melt, Pa·s.

**Figure A1:**
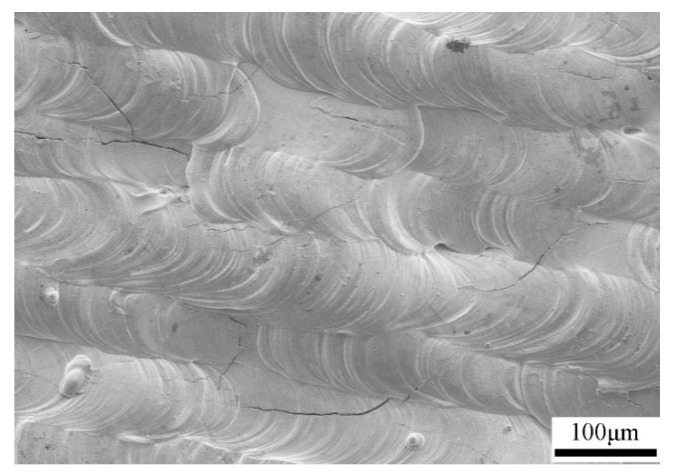
Microcracks on the top surface.

According to the above equations, the surface tension and dynamic viscosity are negatively correlated with the temperature. When the relatively low laser power of 200 W was applied, the energy input was insufficient, accordingly, the heat absorbed by tungsten powder was not enough to fully melt the pre-deposited powder. The temperature of the molten pool formed under the laser power of 200 W was relatively low. In this case, the tungsten melt had a relatively high surface tension and dynamic viscosity. Moreover, the good thermal conductivity of tungsten material provides a favorable condition for heat dissipation. As a result, high the viscosity and fast solidification of tungsten melt hindered the fully spreading of melt before solidifying. Simultaneously, during the laser irradiates tungsten powder, tungsten melt had a more obvious spheroidization tendency due to the high surface tension, and the resultant humps and gaps were formed in the top surface, which can be seen clearly in Figure 4a, illustrated by white arrow and red arrow, respectively. Additionally, the humps or gaps formed on the pre-solidified layer will cause the uniformity of the subsequent powder layer, thus the cavities between adjacent layers will be formed (depicted by the black arrow in Figure 4a) when the laser irradiates the next powder layer. Even worse, the humps with large size may damage the powder scraper, which will lead to the failure of the LPBF process. Furthermore, due to the shrinkage in the solidification process of tungsten melt, the insufficient overlap between adjacent tracks may lead to the formation of shrinkage pores depicted by the yellow arrow in Figure 4c. With the laser power increased to 300 W, the temperature of the molten pool increased rapidly due to enough energy input. As mentioned above, the surface tension and viscosity decreased with elevated temperature. As a consequence, the tungsten melt with high spreading speed and enough spreading time can fully spread before solidifying. Finally, the surface defects can be eliminated, and a relatively dense surface can be obtained, as shown in Figure 4c. The top surface morphology changes obviously with the change of laser power, which implies that the laser power has a pronounced influence on surface morphology. In addition, the surface cracks can be seen clearly from the SEM image (see Figure A1 in the Appendix A). Due to the residual stress caused by high thermal gradient during the LPBF process and inherent brittleness of tungsten material at room temperature as mentioned earlier, cracks were easily formed in the tungsten samples.

**Figure 5:**
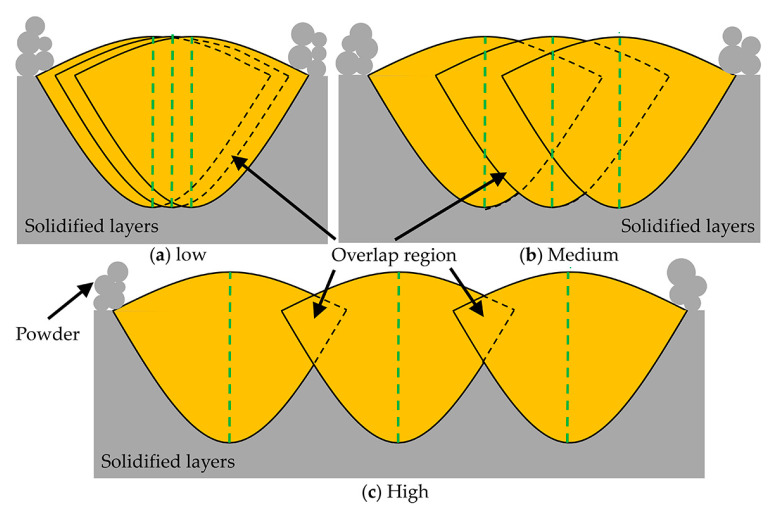
Illustration of different hatch spacings at fixed laser power and scanning speed.

When the laser power of 300 W and hatch spacing of 0.08 mm were applied, Figure 4c–e give the top surface morphology at the scanning speeds of 300 mm/s, 200 mm/s, and 400 mm/s, respectively. It can be observed that when the scanning speed varied from 200 mm/s to 400 mm/s, the top surface morphology remains relatively flat without significant surface defects mentioned above, and only some surface pores exist. Furthermore, the top surface morphology at a scanning speed of 200 mm/s presents some fluctuations and no significant single-track. This can be ascribed to the temperature of the molten pool increased rapidly at low scanning speed, and thus the instability of the molten pool slightly intensified due to overheating tungsten melt. With the increase of scanning speed, the stability of the molten pool improved. As shown in Figure 4e, the relatively flat without significant fluctuation on the surface morphology when the scanning speed increased to 400 mm/s. Figure 4c,f,g present the top surface morphology with different hatch spacing at the fixed laser power of 300W and scanning speed of 400 mm/s. A low hatch spacing means that the overlap region between tracks increased (Figure 5a), thus resulting in multiple re-melting of more regions of solidified tracks, which will intensify the instability of the molten pool. Consequently, the flatness of formed surface morphology decreased slightly, as shown in Figure 4f. Obviously, the flatness of the top surface improved with the increase of hatch spacing, as presented in Figure 4c, which can be explained by appropriate overlap region between neighboring tracks, as illustrated in Figure 5b. It is foreseeable that with the further increase of hatch spacing, the intertrack pores will appear, as shown in Figure 4g. This can be explained by the reduction of overlap region (Figure 5c), the tungsten melt cannot fully fill gaps between the adjacent tracks, which will lead to the appearance of shrinkage pores between the neighboring solidified tracks.

### 3.2. Relative Density

**Figure 6:**
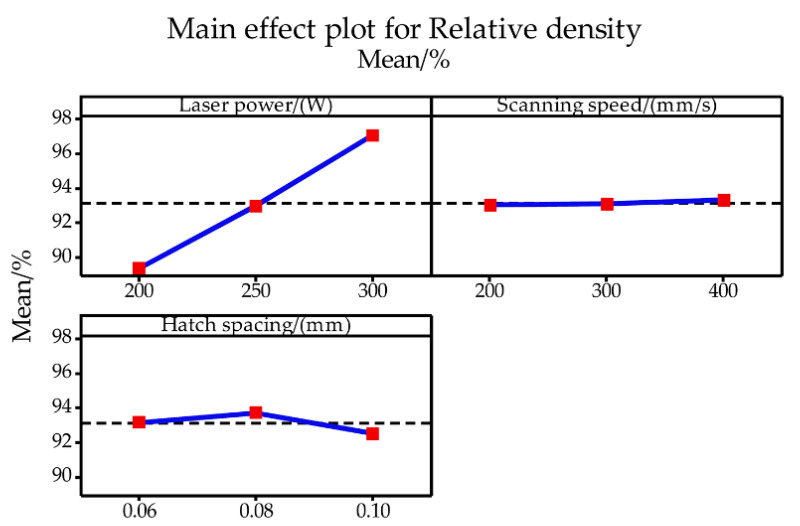
Main effect plots for relative density.

**Figure 7:**
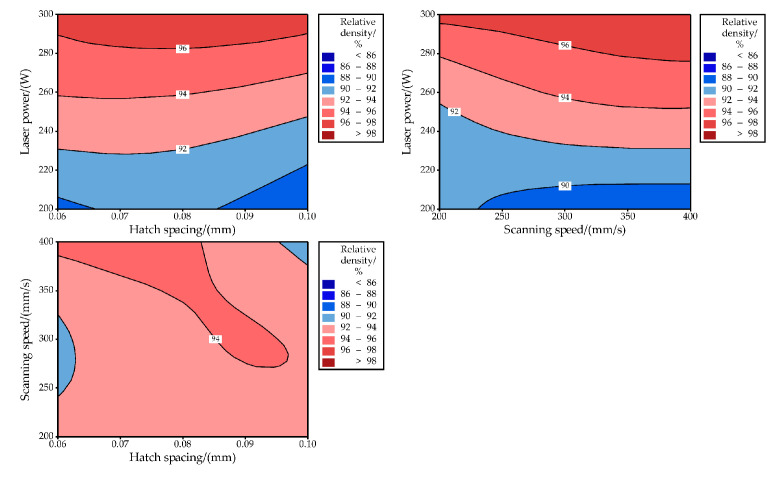
Contour plot showing the influence of process parameters on the RD of as-built W.

In addition to the surface morphology, the relative density of the tungsten samples is also affected by the process parameters. Table 1 lists the measured relative density of as-built tungsten samples for each process parameter combination. Tungsten samples with high density can be manufactured based on the selection of appropriate process parameters, and the maximum relative density was 98.31%. LPBF is a process with multiple factors, and the importance of the process parameters for the relative density is quite different. Figure 6 illustrates the main effect plots, showing the effect of laser power, scanning speed, and hatch spacing on relative density. The plots clearly present the increase of relative density with the higher laser power was applied. And, with the increase of scanning speed, the relative density increases slightly. Meanwhile, the relative density increased slightly and then decreased with the increase of the hatch spacing. Table 2 gives the results of the ANOVA. It can be found that the P-value for the laser power is less than 0.05 indicating that it has a major influence on the variation of final relative density. At the same time, the P-value of 0.939 for scanning speed is significantly higher than 0.05, suggesting a limited influence of scanning speed on the final relative density of as-built tungsten samples. Similarly, the P-value of hatch spacing is 0.409, indicating a relatively low effect of relative density. Naturally, the laser power presents a decisive role in the change of final relative density during the LPBF process of tungsten. Furthermore, according to the analysis results of Table 2, as the dominant influencing factor, the laser power contributed 79.58% to the variation of relative density, while scanning speed and hatch spacing contributed 0.13% and 2.03%, respectively. Additionally, the effects of laser power, scanning speed, and hatch spacing on relative density are also presented by the contour plots as shown in Figure 7. As can be seen in Figure 7, as long as the applied laser power is relatively high, no matter how the scanning speed and hatch spacing change, the tungsten samples with relatively high density (≥90%) can be prepared by LPBF.

**Figure 8:**
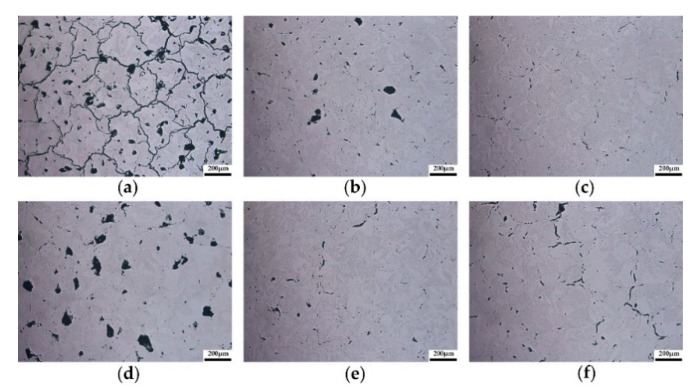
Cross-sectional morphology of different process parameters. (**a**) T8, (**b**) T17, (**c**) T26, (**d**) T1, (**e**) T20, (**f**) T27.

Figure 8 gives the OM images of cross-sectional morphology of as-built tungsten samples prepared using different process parameter combinations. Figure 8a–c present the cross-sectional morphology of as-built tungsten samples under different laser power when a fixed scanning speed of 400 mm/s and hatch spacing of 0.08 mm were applied. It can be found that with the laser power increased from 200 W to 300 W, the inner defects of as-built tungsten samples were greatly reduced. Obviously, the variation of laser power can directly influence the inner defects of as-built tungsten samples and its final relative density, which is consistent with the previous results of ANOVA. The cross-sectional morphology presented in Figure 8c,e, and f can also prove this conclusion. When the fixed laser power of 300 W was applied, although some inner defects appeared, the cross-sectional morphology did not change a lot with the variation of scanning speed and hatch spacing. Besides, by comparing Figure 8a,d, when the laser power was relatively low (200 W); appropriately lowering the scanning speed and hatch spacing can reduce inner defects to a certain extent. As a matter of fact, these phenomena can be explained by the different energy inputs caused by various process parameter combinations. As discussed above, because of insufficient energy input, surface morphology containing various types of defects tends to be formed in the LPBF process. Therefore, the inner defects can be observed in the final as-built tungsten samples due to the layer-by-layer forming characteristics. With the improvement in the morphology of each layer by selecting appropriate process parameters, the inner defects of the tungsten samples can be reduced, thereby increasing its final relative density. Moreover, it is worth pointing out that no matter what process parameters were used, inner microcracks seem to be inevitable. According to the previous studies, large thermal stress tends to be formed in LPBF due to the rapid melting and solidification process, which will cause the formation of microcracks and deformation of parts [27]. Additionally, Vrancken et al. [28] and Wang et al. [29] also proved by experiments and theoretical calculations that microcracks in pure tungsten samples of LPBF were inevitable due to the inherent properties of tungsten.

### 3.3. Microstructure

**Figure 9:**
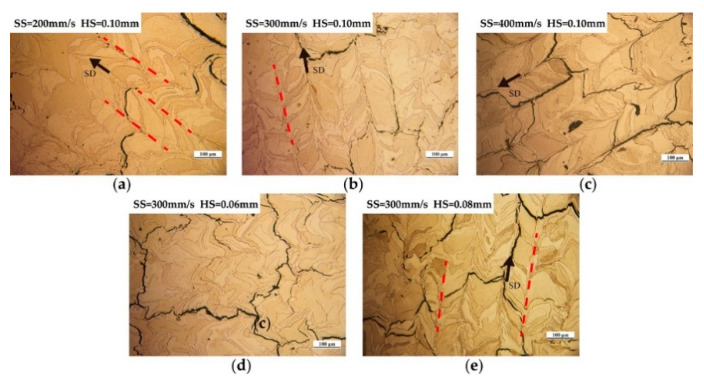
Microstructure of tungsten samples at different process parameters. (**a**) T21, (**b**) T24, (**c**) T27, (**d**) T22, (**e**) T23.

**Figure 10:**
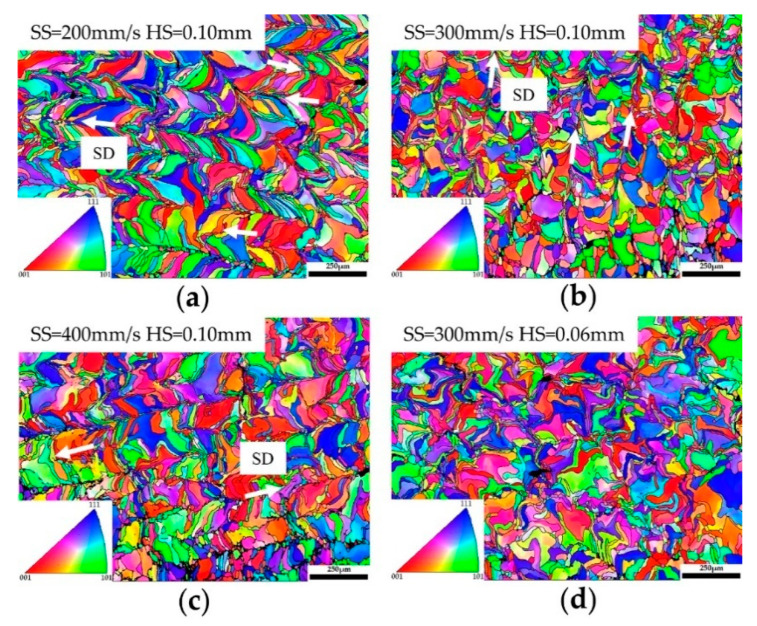
Electron backscattered diffraction (EBSD) images of the cross-section at different scanning speeds and hatch spacing. (**a**) T21, (**b**) T24, (**c**) T27, (**d**) T22.

On the basis of obtaining tungsten samples with a high relative density, the solidified microstructure under different process parameters was characterized and discussed. Figure 9 presents the final microstructure of the as-built pure tungsten samples at different scanning speeds and hatch spacing when the fixed laser power of 300 W was applied. Two types of cracks can be clearly found in Figure 9. Longitudinal cracks tended to form at the center of solidified tracks and grow along the scanning direction (SD), while transverse cracks were generated along the grain boundaries and had a certain inclination angle with the scanning direction. As mentioned earlier, large residual stress in LPBF and the inherent brittleness of tungsten are the reasons that induce the formation of cracks. Figure 9a,b, and c show the typical microstructure of as-built tungsten samples at scanning speed of 200 mm/s, 300 mm/s, and 400 mm/s when the same hatch spacing of 0.10 mm was used. A solidified microstructure can be observed with a distinguishable difference when the applied scanning speed varied from 200 mm/s to 400 mm/s. When the low scanning speed (200 mm/s) was applied, a great number of slender grains formed, which tended to grow along the scanning direction, as depicted in Figure 9a. Figure 10a presents such characteristics of grain growth. It can be observed that the microstructure was almost composed of elongated grains, which grew toward the center of the molten pool in a curved shape. With the increase of scanning speed, as shown in Figure 9b and c, the morphology of the grains changed gradually. The number of elongated grains decreased obviously, and the growth direction of grains is approximately perpendicular to the scanning direction. EBSD image (Figure 10c) further shows the cross-sectional microstructure corresponding to high scanning speed (400 mm/s), a large number of short columnar grains accompanied by few elongated grains can be observed clearly. Besides, according to the EBSD results, the average grain size of as-built tungsten samples decreased from 12.938 μm to 8.977 μm with the scanning speed increased from 200 mm/s to 400 mm/s.

In addition to the scanning speed, the effect of hatch spacing on the microstructure of as-built tungsten samples was also discussed. Figure 9b,d,e give the cross-sectional microstructure under different hatch spacing when the same scanning speed (300 mm/s) was used. As can be seen in Figure 9d, the boundaries of the molten pool became indistinguishable when the low hatch spacing (0.06 mm) was used. With the increase of hatch spacing, the boundaries of the molten pool became distinguishable, as shown in Figure 9b,e. This phenomenon can be attributed to the excessive re-melting between adjacent tracks. In addition, tungsten grains with irregular morphology were distributed randomly in the cross-section and can be observed in both Figure 9d and Figure 10d. The morphology of the tungsten grains did not change significantly with the variations in hatch spacing. Also, the average grain size corresponding to hatch spacing of 0.06 mm, 0.08 mm, and 0.10 mm was 11.914 μm, 12.189 μm, and 10.541μm, respectively. It can be noted that the hatch spacing had no obvious effect on the grain size of as-built tungsten samples.

**Figure 11:**
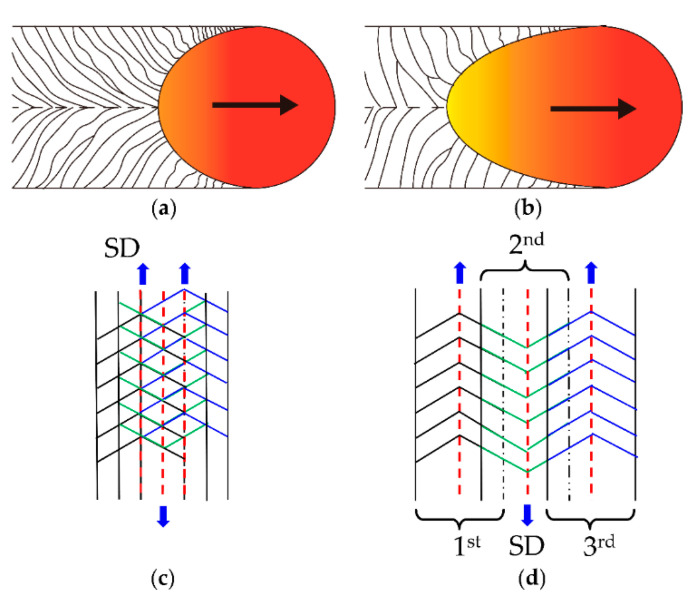
Illustration of grain growth and molten pool morphology under different scanning speeds and hatch spacing. (**a**) low scanning speed, (**b**) high scanning speed, (**c**) low hatch spacing, (**d**) high hatch spacing.

The difference in morphology and size of grains can be explained by different solidification conditions caused by the different process parameters used in the LPBF process. As illustrated in Figure 11a,b, it can be found that the shape of the molten pool shifted from slightly elliptical to teardrop shaped with the increase of scanning speed. During the solidification process, the formed grains tended to grow along the direction of the maximum thermal gradient. Therefore, the relatively straight columnar grains formed at high scanning speed grow toward the centerline of the molten pool while the curved grains formed at low scanning speed grow along the scanning direction. In addition, according to the basic solidification theory, the solidification structure is determined by the thermal gradient and the solidification rate. The solidification rate increased with the increase of scanning speed, thereby the cooling rate increased accordingly, and the resultant solidification grain size decreased. Besides, when the fixed laser power and scanning speed were applied, the basic solidification conditions remain unchanged, thus the grain size did not change significantly. However, the lower hatch spacing means a larger overlapping region between neighboring tracks, as illustrated in Figure 11c. More grains formed in the previous solidified track will be re-melted if the laser irradiates the adjacent powder. Therefore, the molten boundaries disappeared and the final grains with irregular morphology were formed, as shown in Figure 9d and Figure 10d. With the hatch spacing increased further to 0.10 mm, the visible molten pool boundaries were found due to the reduced overlapping region (Figure 11d), while the grain morphology only slightly changed, as illustrated in Figure 9b and Figure 10b.

## 4. Conclusions

This work investigated the surface morphology, relative density, and microstructure of pure tungsten prepared by laser powder bed fusion under different process parameters. The conclusions can be summarized as follows:

1. In terms of surface morphology, a large number of surface defects including gas, humps, cavities, and pores were formed when the low laser power was applied. To obtain a surface with smooth and dense morphology, high laser power should be used in the LPBF process of pure tungsten.
2. The pure tungsten samples with the highest relative density of 98.31% were prepared by using the process parameter combinations: laser power of 300 W, scanning speed of 400 mm/s, hatch spacing of 0.08 mm, and layer thickness of 0.02 mm. Also, compared with scanning speed and hatch spacing, laser power plays a more critical role in the densification process of LPBF for pure tungsten.
3. The typical microstructure of the as-built tungsten sample was characterized and investigated. The elongated curved grains gradually transformed into fine straight columnar grains with the scanning speed increased from 200 mm/s to 400 mm/s, and the average grain size decreased from 12.938 μm to 8.977 μm. Simultaneously, the hatch spacing had a slight influence on the final grain morphology but had no significant effect on the average grain size.
4. The microcracks are inevitable in the tungsten samples fabricated by LPBF. Effective methods of the reduction or elimination of microcracks need to be investigated in the future. Moreover, a relationship between the process parameters and the solidified microstructure can be established in LPBF. Therefore, the microstructure can be tailored to meet different requirements by applying specific process parameters.

## Figures and Tables

**Table 1:** The process parameters used in this work and measured relative density.

No.	LP/(W)	SS/(mm/s)	HS/(mm)	RD/(%)	No.	LP/(W)	SS/(mm/s)	HS/(mm)	RD/(%)
T1	200	200	0.06	91.42	T15	250	300	0.1	94.27
T2	200	200	0.08	91.69	T16	250	400	0.06	95.03
T3	200	200	0.1	89.26	T17	250	400	0.08	95.74
T4	200	300	0.06	86.18	T18	250	400	0.1	90.65
T5	200	300	0.08	90.23	T19	300	200	0.06	95.47
T6	200	300	0.1	90.02	T20	300	200	0.08	96.87
T7	200	400	0.06	90.88	T21	300	200	0.1	97.38
T8	200	400	0.08	88.86	T22	300	300	0.06	96.17
T9	200	400	0.1	85.81	T23	300	300	0.08	97.75
T10	250	200	0.06	92.62	T24	300	300	0.1	97.15
T11	250	200	0.08	90.87	T25	300	400	0.06	98.17
T12	250	200	0.1	91.71	T26	300	400	0.08	98.31
T13	250	300	0.06	92.61	T27	300	400	0.1	96.47
T14	250	300	0.08	93.39	—	—	—	—	—

**Table 2:** Results of analysis of variance for relative density.

Item	Degree of Freedom	Sum of Square	Mean Square	Contributions	F-Value	*p*-Value
LP	2	270.811	133.906	79.58%	43.57	0.000
SS	2	0.424	0.212	0.13%	0.07	0.934
HD	2	6.839	3.419	2.03%	1.11	0.348
Error	20	61.469	3.073	18.28%	–	–
Total	26	336.543	100%	–	–	–

## Data Availability

The data presented in this study are available on request from the corresponding author.
